# When it comes to teaching and tenure it is time to walk the walk

**DOI:** 10.7554/eLife.50542

**Published:** 2019-08-12

**Authors:** Andrew W Murray, Diane K O'Dowd, Chris D Impey

**Affiliations:** 1 Department of Molecular and Cellular BiologyHarvard UniversityCambridgeUnited States; 2 Department of Developmental and Cell BiologyUniversity of California, IrvineIrvineUnited States; 3 Department of AstronomyUniversity of ArizonaTucsonUnited States; eLifeUnited Kingdom

**Keywords:** point of view, teaching, careers in science, promotion and tenure, professors, service

## Abstract

Institutions should value teaching and service, and not just research, when considering faculty for promotion and tenure

Academic scientists are judged in three worlds – research, teaching, and service. In the world of research we create new knowledge, and our colleagues evaluate our performance based on its creativity, rigor, volume, and impact. In the world of teaching we impart knowledge to our students, and they evaluate our performance. And in the world of service, we do work for the communities inside and outside our institution, and are judged on the depth and number of our activities. In the end, these three forms of evaluation are combined to make the decisions about promotion and tenure that impact each faculty member individually, and collectively determine the composition of the faculty at large.

At many research universities, however, decisions on promotion and tenure appear to reflect a belief that research is valued more highly than teaching and service. This view is detrimental to all three activities. If faculty are judged primarily on the basis of their research (Bradforth et al., 2015), we fail the students we teach and the academic communities we belong to (Dennin et al., 2017).

In research, the fastest way of testing whether you really understand something is to teach it to a classroom of non-experts. The process of turning a thicket of poorly organized and sometimes contradictory facts into something comprehensible to students has helped many professors to see new, simpler approaches to aspects of their own research. Moreover, many students are inspired by the fact that their teachers are also involved in creating new knowledge through their research (Freeman et al., 2014).

Student evaluations are the primary means of assessing teaching (Berk, 2005), which is unfortunate because there is ample evidence that such evaluations are flawed tools for measuring teaching effectiveness or student learning. A recent meta-analysis of nearly a hundred studies over forty years finds no significant correlation between a student’s evaluation of teaching and their learning (Uttl et al., 2017). Another concern with student evaluations is that they can reflect biases, including biases related to the gender and/or ethnicity of the teacher (Boring et al., 2016).

There are, however, a variety of other tools that can be used to effectively evaluate different aspects of teaching. These can be grouped into the following four categories: i) student-based, exemplified by student course evaluations; ii) self-reflective, such as statements/portfolios that include learning objectives and specific examples of teaching approaches; iii) peer-based, such as classroom observation by expert faculty; iv) learning gains, ideally using validated instruments for pre- and post-class assessment. None of these methods is perfect, but in combination they can offer a fairer and better integrated view of teaching performance.

The focus should be on identifying and rewarding effective and innovative teaching practice, not about exposing weaknesses.

Why aren't these alternative methods more widely used? The most common reasons are that they take too much work, that teaching is harder to evaluate than research, and the prejudice that good teaching is less intellectually rigorous than good research. Faculty members need models of best practices and support from administrators in implementing a nuanced evaluation of teaching. They also need different modes of feedback from students and peers (Gormally et al., 2014). The focus should be on identifying and rewarding effective and innovative teaching practice, not about exposing weaknesses. Shifting teaching from a solitary pursuit to an activity seen and reviewed by our peers transforms it into a more scholarly activity.

It is over a decade since Handelsman et al. explored "the reasons for the slow pace of change in the way science is taught at research universities" in the United States (Handelsman, 2004), and more recently, one of the present authors (DKO'D) and other Howard Hughes Medical Institute (HHMI) Professors suggested a variety of ways to recognize, reward, and support teaching (Anderson et al., 2011). But teaching will only improve if becoming a good teacher helps with promotion and tenure, and if junior faculty know the criteria for promotion and tenure and how they are applied.

A recent study of promotion and tenure documents and policies at 51 academic institutions in the United States found policy language stating that teaching was valued in the promotion and tenure process at all 51 institutions (Dennin et al., 2017). In addition, 41 of the 51 had some form of guidelines about how teaching should be evaluated, and about half explicitly recommended or required using multiple forms of evidence to evaluate teaching. However, the link between the written policies and actual practice was often weak. We argue that actually following the written policies for promotion and tenure is an essential first step towards increasing the value placed on teaching at research universities. We must start to "walk the walk".

At many research universities, the level of accomplishment necessary for promotion and tenure is represented by summing a faculty member’s contributions to research, teaching, and service (Dennin et al., 2017). In this system, research is often valued more highly than teaching or service, and tenure can be attained by accomplishments in research alone. One solution is to move to a system where a level of accomplishment is required in all three areas: if a candidate does not reach the required level in research, teaching and service, then promotion is denied.

Shifting teaching from a solitary pursuit to an activity seen and reviewed by our peers transforms it into a more scholarly activity.

Finally, even if policies are strengthened, they must be enforced. A practical obstacle is the fact that departmental promotion and tenure committees are skewed toward senior faculty, those least likely to use evidence-based teaching methods or be aware of their efficacy. Distributing responsibility for the evaluation of teaching more broadly among faculty, particularly those with expertise in higher education, would improve the uniformity with which policies are applied. This would, in turn, increase the confidence of faculty that policies are being applied fairly, and have the welcome side effect of bringing scholars in different disciplines together to talk about our core academic values.
